# The Effect of Cholesterol‐Loaded Cyclodextrin and Resveratrol Compounds on Post‐Thawing Quality of Ram Semen

**DOI:** 10.1002/vms3.70172

**Published:** 2025-01-10

**Authors:** Eser Ahmet, Arıcı Ramazan, Yağcıoğlu Selin, Sandal Asiye İzem, Ersoy Nur, Demir Kamber, Evecen Mithat, Ak Kemal

**Affiliations:** ^1^ Department of Reproduction and Artificial Insemination, Faculty of Veterinary Medicine Siirt University Siirt Turkey; ^2^ Department of Reproduction and Artificial Insemination, Faculty of Veterinary Medicine İstanbul University—Cerrahpasa Avcilar İstanbul Turkey; ^3^ Graduate Education Institute Istanbul University—Cerrahpasa Avcılar İstanbul Turkey

**Keywords:** cholesterol, cryopreservation, ram sperm, resveratrol, spermatological examination

## Abstract

Ram sperm are more vulnerable to freezing than those of most other farm animals. During sperm freezing, the cell membrane loses some of its cholesterol, which regulates signalling mechanisms and prevents premature capacitation. Resveratrol (RES) increases the fluidity of the cell membrane, which becomes peroxidized during freezing and reduces free radicals. In this study, the effectiveness of RES, cholesterol‐loaded cyclodextrin (CLC) and their combinations in ram sperm cryopreservation were investigated. The collected semen was divided into two equal volumes: One was diluted with tris‐citric acid‐glucose medium (TCG) containing CLC, whereas the other was diluted with a CLC‐free TCG solution. After examining motility, both groups were further divided into two equal volumes, forming the following working groups: control (no RES, no CLC); RES (20 µg/mL); CLC (2 mg CLC/120 × 10^6^ sperm); and RES + CLC (RES 20 µg/mL + 2 mg CLC/120 × 10^6^ sperm). These groups were diluted with media containing their respective additives. Post‐thawing, the samples were analysed for motility, acrosome and membrane integrity, membrane functionality, mitochondrial activity, capacitation status, oxidative stress and DNA integrity. CLC preserved sperm total motility, acrosome and plasma membrane integrity and decreased the rate of early capacitation (*p* < 0.05). RES had no significant effect on sperm quality before freezing and post‐thawing (*p* > 0.05). However, RES + CLC increased mitochondrial activity post‐thawing (*p* < 0.05). In conclusion, CLC minimized sperm membrane damage caused by cryopreservation in ram sperm. RES alone was ineffective, and the combination of RES and CLC did not yield a positive synergistic effect on ram spermatological parameters.

## Graphical abstract

Cholesterol‐loaded cyclodextrin (CLC) adversely affected the progressive motility of ram sperm during the pre‐freezing period but offered protection during freezing and thawing, whereas 20 µg/mL resveratrol had no beneficial effects; the combination of RES and CLC was more effective than the control but did not demonstrate a synergistic effect.

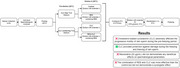

## 1 Introduction

Artificial insemination in domestic animals is the most practical and successful reproductive biotechnology for disseminating desired genetic traits in breeding studies (Vishwanath 2003). Sperm freezing and storage are crucial for preserving genetic resources, enabling use in various regions and accelerating breeding efforts. However, the freezing process induces structural, biochemical and functional changes in sperm, potentially damaging parameters such as motility, viability and morphological integrity (Uysal and Bucak 2009).

The membrane cholesterol/phospholipid ratio regulates membrane fluidity during freezing and reduces cell damage from thermal shock. In ram sperm, this ratio is less than 0.37, making them more susceptible to freezing compared to other mammals such as bulls, dogs and humans. Additionally, during sperm freezing, some cholesterol is lost from the cell membrane, disrupting signalling mechanisms and inducing early capacitation (Maldjian et al. 2005). This can also cause a premature acrosome reaction, leading to a loss of fertilizing ability (Langlais and Roberts 1985).

In recent years, researchers have added exogenous cholesterol to extenders to increase the cholesterol/phospholipid ratio in ram sperm. As pure cholesterol is slightly soluble in water, it is recommended to use cholesterol‐loaded vehicles in the diluent. Cyclodextrins, cyclic oligosaccharides with external hydrophilic and internal hydrophobic cavities, are widely used for this purpose (Collin et al. 2000). They have a high affinity for steroids and, when preloaded with cholesterol (cholesterol‐loaded cyclodextrin [CLC]), facilitate the incorporation of cholesterol into cell membranes. This helps maintain the cholesterol/phospholipid ratio in the sperm plasma membrane during cryopreservation, reducing cryocapacitation‐like alterations and programmed cell death (Nain et al. 2023). Recent research has focused on using exogenous CLC in extenders to increase the cholesterol/phospholipid ratio and protect the sperm cell membrane from freezing damage (Sharafi et al. 2022; Navratil et al. 2003; Moore, Squires, and Graham 2005; Purdy and Graham 2004; Rajoriya et al. 2016; Katanbafzadeh, Barati, and Tabandeh 2014; López‐Revuelta et al. 2006).

Another factor that damages the structural and physiological features of sperm is the formation of reactive oxygen species (ROS) and oxidative degeneration during the freezing stage (Watson 2000; Sarlos, Molnar, and Kokai 2002). Although several antioxidant systems are naturally present in both seminal plasma and intracellular settings, the ROS protection they provide during the freezing process is insufficient. Resveratrol (RES) is a natural polyphenol produced by many plant species to defend against stress, UV radiation and fungal diseases (Savouret and Quesne 2002). It is a potent antioxidant that suppresses the generation of free radicals such as superoxide anion, hydroxyl radicals and metal‐induced radicals (Leonard et al. 2003). RES has stronger antioxidant activity than major antioxidants like vitamins E and C (Stojanović, Sprinz, and Brede 2001), with less severe detrimental effects (Juan et al. 2005; Kyselova et al. 2003), and can be employed as an antioxidant in the cryopreservation of ram sperm (Sarlos, Molnar, and Kokai 2002; Al‐Mutary 2021). Studies have indicated that the inclusion of RES in extenders during the cryopreservation of ram semen activates antioxidant systems in sperm, mitigates oxidative stress induced by ROS and enhances sperm quality (Zhu et al. 2023; Silva et al. 2012; Brair et al. 2024; Chen et al. 2024). Furthermore, studies on various species, including bull (Li et al. 2018; Bucak et al. 2015; Assunção et al. 2021; Correa et al. 2024; Sharafi et al. 2022), buffalo (Longobardi et al. 2017; Ahmed et al. 2020), goat (Lv et al. 2019; Falchi et al. 2020), pig (Zhu et al. 2019; Kaeoket and Chanapiwat 2023), stallion (Giaretta et al. 2014) and human sperm (Garcez et al. 2010; Shabani Nashtaei et al. 2017; Branco et al. 2010), have demonstrated that RES has beneficial effects on several spermatological parameters following cryopreservation.

This study aimed to investigate the potential benefits of dual protection (preventing membrane peroxidation and maintaining membrane stabilization) on post‐thawed ram sperm using CLC and RES, either separately or in combination, during cryopreservation.

## 2 Materials and Methods

This study was conducted out of the breeding season (March–June). The materials were five Kıvırcık rams (*n* = 5) aged 2–5 years, housed at Istanbul University‐Cerrahpasa Faculty of Veterinary Medicine. During the study, the rams were maintained under standard management and feeding protocols, and they were housed separately from other animals.

### 2.1 Preparation of Semen Extenders

All chemicals used in this study, unless otherwise specified, were purchased from Sigma Chemical Co. (Saint Louis, MO, USA).

Four experimental groups were established: control (no CLC, no RES); RES (RES 20 µg/mL); CLC (2 mg CLC per 120 × 10^6^ sperm); RES + CLC (RES 20 µg/mL + 2 mg CLC per 120 × 10^6^ sperm). The sperm dilution rate was 50 × 10^6^ motile sperm/straw. In each experimental trial, 20 straws were frozen for each group.

Tris‐based egg yolk medium was used as the base semen diluent in all groups (Cirit et al. 2013). The extender was split into two equal volumes (extenders A and B), and 10% glycerol was added to extender B.

#### 2.1.1 CLC Preparation

The CLC (methyl‐β‐cyclodextrin; Cat. no: C4555, cholesterol; Cat. no: C8667) used in this experiment was applied at a concentration of 2 mg of CLC per 120 × 10^6^ sperm, as described for sheep by Mocé, Purdy, and Graham (2010). The method described by Purdy and Graham (2004) was used for the production of CLC. A tris‐citric acid‐glucose (TCG) solution [25 mM Tris (Cat. no: T6066), 8 mM citric acid (Cat. no: C2404) and 7 mM glucose (Cat. no: F3510)] was prepared to dissolve CLC. Powdered CLC (50 mg/mL) was dissolved in the TCG solution, and 5 mg/mL bovine serum albumin (BSA, Cat. no: A3311) was added.

#### 2.1.2 Preparation of Extender Containing RES

In this study, 20 µg/mL of RES (Cat. no: R5010) was used, similar to a previous study (Silva et al. 2012) that demonstrated the effectiveness of adding RES to semen extenders for ram sperm cryopreservation. Twenty‐five milligrams of RES was dissolved in 1 mL of dimethyl sulfoxide (DMSO, Cat. no: 276855) for accurate dosing. The solution was divided into 20 µL aliquots and stored at −20°C. On experiment days, a thawed 20 µL aliquot was mixed with 80 µL of Tris‐based egg yolk base extender and vortexed.

Twenty microlitres of extender A was removed and replaced with an equal volume of DMSO containing RES in Tris‐based egg yolk medium.

### 2.2 Cryopreservation Protocol

**FIGURE 1:**
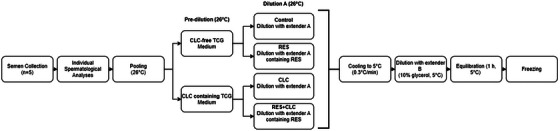
Schematic representation of experimental procedures and sperm cryopreservation protocol. CLC, cholesterol‐loaded cyclodextrin; RES, resveratrol; TCG, tris‐citric acid‐glucose.

Semen samples were collected using an artificial vagina. The spermatological parameters of each ram were analysed individually, and only samples having specific quality criteria (mass motility: ≥+++; motility: ≥80%; volume: ≥0.5 mL; sperm concentration: ≥1 × 10^9^/mL) were pooled (1 mL from each ram's ejaculate) to minimize individual variations (Cirit et al. 2013). Sperm motion parameters and concentration were assessed after pooling using a Computer‐assisted Sperm Analysis (CASA 12.3 IVOS, Hamilton, USA) system. The semen volume containing 4 × 10^9^ motile sperm was divided into two equal portions. The first portion was diluted with TCG containing CLC (2 mg/120 × 10^6^ sperm). The other portion was diluted with an equal volume of CLC‐free TCG solution. After dilution, the samples were incubated for 15 min in a 26°C water bath. At the end of the incubation period, total and progressive motility assessments were conducted. Subsequently, both CLC and CLC‐free groups were divided into two equal volumes to form the treatments groups (control, RES, CLC and CLC + RES), as depicted in Figure 1. All groups were gradually diluted with extender A in a 26°C water bath. For the CLC and CLC + RES groups, 20 µL of extender A was removed and replaced with an equal volume of DMSO + Tris‐based egg yolk medium.

The groups diluted with extender A were cooled to 5°C at a rate of 0.3°C/min using a controlled‐rate freezer (Bio‐cool III). At this temperature, glycerolization (dilution with extender B) was performed gradually, adding 10%, 20%, 30% and 40% of extender at 8‐min intervals (Shabani Nashtaei et al. 2017). After dilution, the final sperm concentration reached 200 × 10^6^ sperm/mL, with 5% glycerol. Subsequently, all groups were incubated at 5°C for 1 h to equilibrate, and sperm total and progressive motility parameters were evaluated using CASA at the end of the incubation period. Samples were then loaded into mini straws and subjected to freezing in N_2_ vapor. After 10 min, the straws were transferred into liquid nitrogen (LN_2_) for storage until spermatological examinations (Ak et al. 2010).

### 2.3 Spermatological Examinations

#### 2.3.1 CASA Examinations

The CASA examinations were performed three times: after the addition of the TCG (CLC and CLC‐free), after equilibration and after thawing periods. Before conducting the examinations, the CASA software was calibrated according to the guidelines in the IVOS Version 12.3 user manual (Demir et al. 2015).

#### 2.3.2 Hypoosmotic Swelling Test (HOST)

The functional integrity of sperm plasma membranes was assessed using a modified hypoosmotic swelling (HOS) test. Spermatozoa that reacted to the hypoosmotic environment by bending, coiling or shortening their tails were considered to have functional plasma membranes (Bacinoglu et al. 2008).

#### 2.3.3 Assessment of Capacitation Status

Fluorescent chlortetracycline (CTC, Cat. no: C4881) dye was utilized to assess the capacitation status of spermatozoa. A working solution was prepared by dissolving 2 mg of CTC powder in 5 mL of a Tris‐based diluent [20 mM Tris (Cat. no: T6066), 130 mM NaCl (Cat. no: S5886), 5 mM cysteine (Cat. no: C7352)]. Then, 10 µL of semen and 15 µL of the CTC working solution were added to a microcentrifuge tube. The samples were incubated at room temperature for 5 min, after which 0.5 µL of Hancock solution was added to stop the reaction. Three microliters of the sample was placed on a slide, covered with a coverslip and evaluated by counting 100 cells at 400× magnification using a fluorescence microscope (Eclipse Ni‐U, Nikon, Tokyo, Japan) with 485/20 nm excitation and 580–630 nm emission filters. Sperm with fluorescence localized to the acrosomal and post‐acrosomal regions of the head were categorized as non‐capacitated (F pattern), whereas those showing fluorescence throughout the head were considered capacitated (Perez et al. 1996).

#### 2.3.4 Spermatological Parameters Examined by Flow Cytometry

##### 2.3.4.1 Assessment of Plasma Membrane Integrity

The protocol was modified from the staining method described by Câmara et al. (2011). The thawed sperm sample was diluted with a Tris‐based solution to achieve a concentration of 50 × 10^6^ sperm/mL. Then, 0.5 µL of carboxyfluorescein diacetate (CFDA, Cat. no: C5041; 0.46 mg/mL stock), 0.5 µL of propidium iodide (PI, Cat. no: 81845; 0.5 mg/mL stock) and 200 µL of a Tris‐based solution were sequentially added to 100 µL of diluted semen in the dark. The stained samples were subsequently loaded into 96‐well plates. Analysis was conducted by counting 5000 cells using flow cytometry (Guava easyCyte, Guava Technologies, Hayward, CA, USA), with excitation wavelengths ranging from 519 to 630 nm.

The evaluation was conducted by identifying sperm that exhibited green fluorescence without any red fluorescence stimulation (CFDA+, PI−). Spermatozoa falling into this category were classified as ‘viable and possessing an intact plasma membrane’.

##### 2.3.4.2 Evaluation of Acrosome Integrity

The analysis utilized a combination of fluorescein isothiocyanate conjugated to *Arachis hypogaea* (peanut) lectin (FITC‐PNA, Cat. no: L7381) and PI (Cat. no: 81845) dyes, following the protocol described by Marco‐Jiménez et al. (2005) with certain modifications. Briefly, the thawed sperm samples were diluted with a Tris buffer solution to a concentration of 1 × 10^6^ spermatozoa per millilitre and centrifuged at 700 rpm for 5 min. After removing the supernatant, 1000 µL of Tris buffer solution was added. Then, 100 µL of the samples were taken, and 2 µL of FITC‐PNA (100 µg/mL in saline solution), 200 µL of Tris buffer solution and 2 µL of PI (0.5 mg/mL in distilled water) were added in the dark. The samples were incubated at room temperature for 10 min. Finally, 300 µL of the stained samples were transferred to 96‐well plates. Measurements were conducted using flow cytometry to determine the percentage of sperm exhibiting green and/or red fluorescence excitation within the emission range of 519–590 nm.

##### 2.3.4.3 Evaluation of Sperm Mitochondrial Activity

Sperm mitochondrial activity level was measured with the 5,50,6,60‐tetrachloro‐1,10,3,30‐tetraethylbenzimidazolyl‐carbocyanine iodide (JC‐1, Cat. no: T4069) staining protocol. The post‐thawed semen (0.5 mL) was diluted to a concentration of 50 × 10^6^ sperm/mL using a Tris‐based buffer medium. Following this, 200 µL of Tris‐based buffer medium and 0.5 µL of JC‐1 stock solution (3 mM JC‐1 in DMSO) were added to 100 µL of the diluted sample, respectively. The stained sperm samples were then incubated for 40 min in a water bath at 38°C. Following incubation, 300 µL of each sample was transferred into 96‐well microplates. Spermatozoa with orange fluorescence excitation in flow cytometry were evaluated as having ‘high mitochondrial membrane potential (HMMP)’ (Gillan, Evans, and Maxwell 2005).

##### 2.3.4.4 Measurement of Oxidative Stress

Measurements were conducted to quantitatively assess superoxide radicals in spermatozoa subjected to oxidative stress. The presence of ROS in the post‐thaw sperm samples was determined utilizing the Muse Oxidative Stress Kit (Cat. no: MCH100111, Millipore, Bedford, MA, USA). The thawed sperm sample was held at 37°C for 30 s, pooled and diluted to a concentration of 100 × 10^6^ sperm/mL. Then, 190 µL of the kit solution was added to 10 µL of the sample. The prepared samples were placed in 96‐well microplates and analysed using flow cytometry. The proportion of sperm exhibiting red fluorescence excitation (ROS+) was detected and recorded.

##### 2.3.4.5 Terminal Deoxynucleotidyl Transferase dUTP Nick End Labelling (TUNEL) Assay

Sperm deoxyribonucleic acid (DNA) integrity was assessed using the Guava TUNEL kit (Cat. no: 4500‐0121, Millipore, Bedford, MA, USA) following the manufacturer's protocol. In brief, sperm samples were fixed in 4% paraformaldehyde in PBS (pH 7.4) for 1 h at 4°C and subsequently permeabilized with 1% Triton in PBS for 30 min at 4°C. Following fixation and permeabilization, the samples were washed with the washing buffer provided in the kit and incubated in a DNA labelling mix for 2 h at 37°C. After this incubation, the spermatozoa were centrifuged at 300 × *g* for 5 min in 200 µL of rinsing buffer and then incubated in the anti‐BrdU staining mix for 30 min at room temperature in the dark. Finally, 150 µL of rinsing buffer was added, and TUNEL analysis was performed by counting 5.000 cells using flow cytometry. Sperms exhibiting red fluorescent expression were identified as TUNEL+ spermatozoa (Feitosa et al. 2010).

### 2.4 Statistical Analysis

The present study comprises experiments that were repeated 12 times (*n* = 12). SPSS Version 13.0 for Windows (SPSS Inc., Chicago, IL, USA) was employed to evaluate all spermatological parameters. The Student *t*‐test was used to compare the motility data between the CLC and CLC‐free groups. The Kruskal–Wallis test was applied to analyse the data obtained from the TUNEL examination. The one‐way analysis of variance (ANOVA) test was conducted for the statistical analysis of other spermatological examination results, followed by the Tukey HSD test for post hoc comparison of means. The results are presented as mean ± standard error (SE). Differences with *p* values less than 0.05 were considered statistically significant (*p* < 0.05).

## 3 Results

The total motility and progressive motility values of the groups after the addition of TCG (CLC and CLC‐free) solutions to fresh semen are presented in Table 1. There were no statistically significant differences in total motility values between the groups (*p* > 0.05). However, for progressive motility values, the CLC‐free group showed a significantly higher percentage (*p* < 0.05). The presence of CLC in the extender was found to negatively affect sperm progressive motility (*p* < 0.05), as shown in Table 2.

**TABLE 1:** Total motility (%) and progressive motility (%) values (computer‐assisted sperm analysis [CASA]) after dilution of fresh semen samples with cholesterol‐loaded cyclodextrin (CLC) and CLC‐free Tris‐citric acid‐glucose (TCG) solution (*n* = 12).

Groups	Total motility (%)	Progressive motility (%)
**CLC**	95.41 ± 1.12	58.25 ± 1.95^b^
**CLC‐free**	95.16 ± 1.20	68.08 ± 1.57a

*Note*: Different literals within columns mean statistical differences.

Abbreviation: CLC, cholesterol‐loaded cyclodextrin.

^ab^ *p* < 0.05.

**TABLE 2:** Total motility (%) and progressive motility (%) values determined by computer‐assisted sperm analysis (CASA) after equilibration (*n* = 12).

Groups	Total motility (%)	Progressive motility (%)
Control	89.08 ± 2.70	48.66 ± 3.79^a^
RES	95.25 ± 1.64	49.25 ± 2.96^a^
CLC	91.83 ± 1.74	35.41 ± 3.11^b^
RES + CLC	92.25 ± 1.12	34.91 ± 3.53^b^

*Note*: Different literals within columns mean statistical differences.

Abbreviations: CLC, cholesterol‐loaded cyclodextrin; RES, resveratrol.

^ab^ *p* < 0.05.

After thawing, the groups containing CLC exhibited improved total motility (*p* < 0.05) (Table 3). Additionally, kinetic motion parameter values were higher in the groups containing CLC (CLC group, RES + CLC group) after thawing (*p* < 0.05) (Table 3). The RES group, which had the highest percentage of static sperm at 36.83% ± 2.86%, demonstrated a significant decrease in sperm motility after thawing (*p* < 0.05) (Table 3). All groups showed statistically similar results (*p* > 0.05) regarding the number of spermatozoa that responded positively to the HOST, indicating functional membrane integrity. CLC inhibited early capacitation in ram sperm (*p* < 0.05), whereas 20 µg/mL RES did not significantly affect the pre‐capacitation sperm rate (*p* > 0.05) (Table 4).

**TABLE 3:** Post‐thaw total motility (%), progressive motility (%), static (%) and kinetic motion parameters (µm/sec) determined by computer‐assisted sperm analysis (CASA) (*n* = 12).

Groups	Total motility (%)	Progressive motility (%)	Static (%)	VAP (µm/s)	VSL (µm/s)	VCL (µm/s)
**Control**	55.27 ± 2.56^b^	34.19 ± 1.65	29.97 ± 2.34^ab^	113.81 ± 1.95^a^	101.82 ± 1.84^a^	174.20 ± 3.19^a^
**RES**	53.13 ± 2.84^b^	31.80 ± 1.60	36.83 ± 2.86^a^	112.81 ± 1.90^a^	100.35 ± 1.56^a^	173.43 ± 3.83^a^
**CLC**	74.19 ± 1.87^a^	35.00 ± 1.27	18.08 ± 1.95^c^	87.88 ± 1.16^b^	74.83 ± 0.95^b^	140.13 ± 2.04^b^
**RES + CLC**	69.55 ± 2.64^a^	32.27 ± 1.47	22.72 ± 2.73^cb^	87.55 ± 0.91^b^	74.75 ± 0.92^b^	139.60 ± 1.27^b^

*Note*: Different literals within columns mean statistical differences.

Abbreviations: RES, resveratrol; VAP, path speed; VCL, curvilinear velocity; VSL, progressive velocity.

^abc^ *p *< 0.05.

**TABLE 4:** Plasma membrane integrity (%), acrosome integrity (%), high mitochondrial activity (%), oxidative stress (%), DNA fragmentation (%), hypoosmotic swelling test (%) and non‐capacitated (F pattern) sperm (%) values after thawing (*n* = 12).

Groups	Plasma membrane integrity (%)	Acrosome integrity (%)	High mitochondrial activity (%)	Oxidative stress (%)	DNA fragmentation (%)	Hypoosmotic swelling test (%)	Non‐capacitated sperm (F pattern) (%)
**Control**	29.49 ± 1.63^b^	24.16 ± 1.80^b^	42.15 ± 5.18^b^	51.37 ± 5.39^b^	4.99 ± 1.37	38.66 ± 1.36	35.25 ± 0.91^c^
**RES**	26.86 ± 1.84^b^	19.81 ± 2.34^b^	48.51 ± 2.30^b^	63.01 ± 3.90^a^	3.67 ± 0.89	39.83 ± 1.03	36.83 ± 0.86^c^
**CLC**	54.79 ± 1.85^a^	55.09 ± 1.88^a^	53.85 ± 5.51^b^	47.11 ± 3.11^b^	2.81 ± 0.43	40.05 ± 1.68	59.16 ± 0.90^a^
**RES + CLC**	53.60 ± 2.63^a^	53.55 ± 2.96^a^	70.85 ± 2.84^a^	45.49 ± 3.21^b^	3.31 ± 0.65	41.75 ± 1.20	50.41 ± 0.98^b^

*Note*: Different literals within columns mean statistical differences.

Abbreviations: CLC, cholesterol‐loaded cyclodextrin; RES, resveratrol.

^abc^ *p *< 0.05.

The data from the post‐thaw flow cytometry analyses are presented in Table 4. The highest rates of cell membrane integrity and acrosome integrity were observed in the CLC and RES + CLC groups (*p* < 0.05). Additionally, the RES + CLC group exhibited the highest mitochondrial activity rate (*p* < 0.05). The RES group showed significantly higher ROS values compared to the other groups (*p* < 0.05). The DNA fragmentation rates after thawing were similar across all groups (*p* > 0.05).

## 4 Discussion

CLC has a high affinity for lipids in its surroundings. Adding CLC to sperm in a medium containing egg yolk transfers most of its cholesterol to the egg yolk lipids rather than to the sperm. Therefore, incubating sperm with CLC in a lipid‐free medium during cryopreservation is recommended to enhance its effectiveness (Purdy and Graham 2004).

Purdy and Graham (2004) reported that similar benefits were observed with both 15‐ and 60‐min pre‐incubation periods in their study investigating the effect of CLC‐sperm incubation time on post‐thaw motility and viability in bull sperm. They concluded that cholesterol transfer from cyclodextrin to spermatozoa was rapid, with sufficient transfer occurring within just 15 min. In the present study, this rapid interaction may explain the lower proportion of progressive motility observed in the CLC groups after pre‐dilution.

Despite finding that CLC did not affect total motility based on data obtained after equilibration (*p* > 0.05), it was evident during the pre‐dilution process that CLC adversely affected the mechanism of sperm motion, significantly reducing the rate of progressive motility (*p* < 0.05). However, the specific mechanism responsible for the decrease in sperm motility remains unexplained, and further scientific investigation is needed to elucidate this phenomenon.

The CASA results obtained after thawing indicated that sperm total motility and certain kinetic motion properties were preserved more effectively against the adverse effects of the cryopreservation in the CLC groups (*p* < 0.05). This study corroborates post‐thaw motility findings reported in various studies involving bulls (Mocé et al. 2014; Mocé and Graham 2006), rams (Batissaco et al. 2020; Purdy et al. 2010) and goats (Mocé et al. 2014) with the addition of CLC to semen. When the progressive motility rates were examined, very similar results were obtained before freezing and after thawing in the groups containing CLC. The negative effect of CLC on progressive motility observed in the pre‐freezing period was not present after thawing. In the other CLC‐free groups (control, RES), progressive motility rates decreased significantly after thawing. CLC decreased kinetic movement parameter (VAP, VCL, VSL) values and immotile spermatozoa rates after thawing (*p* < 0.05). Because sperm movement was slower in the CLC groups, it can be inferred that cells preserved their energy better, releasing fewer metabolic residues into the environment. Consequently, the presence of CLC may be beneficial in preserving the motion parameters and potential fertility of sperm.

The integrity of the sperm plasma membrane is crucial for capacitation, acrosome reaction, viability maintenance and sperm attachment to the oocyte surface. Therefore, damage to the sperm membrane during the freezing–thawing processes of semen is widely recognized as the primary cause of low fertility rates (Valcarcel et al. 1997). In the HOS test, no statistically significant difference was observed between the experimental and control groups (*p* > 0.05). However, higher rates were detected in the CLC and CLC + RES groups using the CFDA‐PI staining method (*p* < 0.05). The findings from numerous studies investigating the effect of CLC on protecting the sperm membrane support the positive results obtained in our study (Mocé, Purdy, and Graham 2010; Purdy and Graham 2004; Câmara et al. 2011; Batissaco et al. 2020; Inanc, Uysal, and Ayhan 2018; Elshamy et al. 2019). However, it is worth noting that the addition of CLC did not influence the functional integrity of the sperm membrane (*p* > 0.05). Studies conducted on model cell membranes have demonstrated that increased cholesterol concentrations lead to reduced membrane permeability. The addition of cholesterol decreases membrane fluidity, thereby increasing the tightness of membrane components and reducing the passive passage of water and molecules through the membrane (Müller et al. 2008). Therefore, in a hypoosmotic environment, the passage of water into the sperm membrane may be reduced, potentially resulting in a decreased reaction rate of sperm belonging to CLC groups in the HOS test.

It is well established that a correlation exists between mitochondrial activity and sperm motility. Nonetheless, the lifespan of sperm is inevitably diminished due to inadequate energy resources and heightened mitochondrial activity (Ma et al. 2011). Research findings regarding the impact of CLC on sperm mitochondrial activity are inconsistent. Some studies have indicated that CLC does not influence sperm mitochondrial activity (Inanc, Uysal, and Ayhan 2018; Spizziri et al. 2010). However, another study on ram sperm demonstrated that adding CLC and trehalose to the extender increased the proportion of sperm exhibiting high mitochondrial activity (Mocé and Graham 2006). The mitochondrial system of sperm is the primary source of intracellular ROS generation (Turrens 2003). RES has been reported to reduce mitochondrial ROS generation in somatic cells (Pervaiz and Holme 2009). RES, when added to the medium during the cryopreservation of bull (Bucak et al. 2015), buffalo bull (Ahmed et al. 2020) and pig (Zhu et al. 2019) semen, has been reported to increase sperm mitochondrial activity after thawing. Conversely, another study observed that RES added to ram semen decreased the rate of mitochondrial activity after thawing (Silva et al. 2012). In our study, the mitochondrial activity rates in the control, RES and CLC groups were found to be similar. However, it was observed that the combination of RES and CLC increased mitochondrial activity through a synergistic effect. Despite this increase in mitochondrial activity, there was no corresponding effect on sperm motility. The variation in results across studies could be attributable to the dose of antioxidant used and the extender composition (Bucak et al. 2015). Furthermore, the efficacy of phenolic compounds depends on the administration technique, as well as the type and half‐life of the radicals present (Sies 1993).

Oxidative and osmotic stress experienced during cryopreservation can cause DNA damage in mammalian sperm. Oxidative stress, in particular, is the primary cause of sperm membrane and DNA damage (Glazar et al. 2009). To mitigate these stresses and enhance sperm quality post‐thawing, cholesterol and antioxidant substances are added to semen extenders (Ezz et al. 2017). In the present study, although the percentage of DNA fragmentation in the control group was higher than in the other groups, no statistically significant difference was observed between groups (*p* > 0.05).

It has been reported that CLC supports the continuity of sperm membrane stability and improves semen quality in bulls (Purdy and Graham 2004; LaVelle, Cairo, and Barfield 2023), rams (Mocé, Purdy, and Graham 2010; Purdy et al. 2010), stallions (Spizziri et al. 2010), buffaloes (Longobardi et al. 2017) and donkeys (Oliveira et al. 2014). This improvement is associated with a decrease in the early capacitation rate. The data obtained in the present study were compatible with these studies. Additionally, although the positive effects of ROS on capacitation, hyperactivation, acrosome reaction and sperm‐oocyte fusion are recognized, high intracellular ROS levels can also induce an early acrosome reaction by triggering spermatozoa capacitation. Longobardi et al. (2017) reported that RES decreased the rate of early capacitation in buffalo bull spermatozoa. They proposed that RES reduces the levels of ROS in the environment and inhibits protein tyrosine phosphorylation, which is regulated in the presence of ROS, potentially explaining its role in inhibiting sperm pre‐capacitation. Eser et al. (2024) used a different method from the one employed in the present study, directly incubating ram sperm with 20 µg/mL RES for 15 min and found that early capacitation was inhibited. In contrast, this study found that 20 µg/mL RES in the extender did not prevent early capacitation in ram sperm after thawing (*p *> 0.05).

RES is an indirect activator of SIRT1 (Morita et al. 2012). The addition of RES to sperm extenders has been shown to activate SIRT1 expression, enhance the activity of glutathione peroxidase (GPx), catalase (CAT) and superoxide dismutase (SOD), and reduce ROS, lipid peroxidation (LPO) and DNA damage in ram sperm. Furthermore, RES has been reported to increase AMPK phosphorylation levels by activating SIRT1, improving mitochondrial function, adenosine triphosphate (ATP) content and sperm motility. However, the effect of RES on ram sperm is dose‐dependent, with the optimal dose being 50 µM, whereas higher doses have detrimental effects (Zhu et al. 2023). Nicotinamide adenine dinucleotide (NADH) dehydrogenase 1 beta subcomplex subunit 9 (NDUFB9) is an accessory subunit of the mitochondrial respiratory chain NADH dehydrogenase (complex I), and its downregulation is linked to reduced complex I function and increased mitochondrial ROS accumulation (Wu et al. 2018). Dihydrolipoamide dehydrogenase (DLD) is an energy‐related protein. Inhibition of DLD activity leads to a suppression of the tricarboxylic acid cycle, reduction in NADH production, decreased ATP production through oxidative phosphorylation and consequently reduced sperm viability (Li et al. 2016). The research findings indicate that the addition of 15 mmol/L RES during cryopreservation of ram semen enhances sperm viability through increased levels of DLD and NDUBF9 proteins (Chen et al. 2024). Bucak et al. (2015) reported positive effects of RES on motility, DNA integrity and mitochondrial activity after thawing in bull semen. In human sperm, RES has been reported to reduce DNA damage during the post‐thaw period (Garcez et al. 2010). Certain studies have reported positive effects of RES on acrosome integrity (Silva et al. 2012; Bucak et al. 2015; Kaeoket and Chanapiwat 2023; Eser et al. 2024). Conversely, other studies, including those by Lv et al. (2019), Ahmed et al. (2020), Zhu et al. (2019), Assunção et al. (2021) and Eser et al. (2024), have demonstrated no significant effects. Silva et al. (2012) reported that RES reduced mitochondrial activity but did not affect progressive motility, membrane integrity, acrosome integrity and viability in ram semen. Similarly, in another study, RES was not effective in protecting sperm membrane integrity after thawing in rams (Brair et al. 2024; Eser et al. 2024). On the other hand, there are studies demonstrating that RES reduces the rate of premature capacitation in both buffalo and ram sperm (Brair et al. 2024; Longobardi et al. 2017; Eser et al. 2024). In contrast to the findings, the present study did not observe any visible improvement in semen quality during both pre‐freezing and post‐freezing analyses. The discrepancies observed between studies may stem from several factors, including the species of animals used, the composition of extenders, the dosage and method of RES administration, the specific type of ROS involved and the concentration of frozen spermatozoa per millilitre. These variables can influence the outcomes related to sperm quality and the effects of RES on sperm physiology.

Quantitative measurement of intracellular ROS was performed to determine the oxidative stress level in spermatozoa. It was an unexpected result that the amount of ROS was found to be higher in the RES group compared to the other groups (*p* < 0.05), and these data do not support the results of Longobardi et al. (2017) with buffalo semen. Given that the assessment of oxidative stress focused on detecting intracellular superoxide radicals, RES was found to be ineffective in mitigating these radicals in ram spermatozoa. Therefore, it was concluded that to accurately assess the impact of RES on oxidative stress in ram spermatozoa, analyses capable of detecting various intracellular ROS as well as membrane LPO should be conducted. When ram sperm was directly treated with H_2_O_2_, it is reported that CLC protected ram sperm from ROS. This protective effect was attributed to CLC's antioxidant properties or its ability to maintain membrane stability during cryopreservation stages (Naseer et al. 2015). According to Benhenia et al. (2016), CLC and CLC + vitamin E added to the extender reduced the quantity of LPO in ram sperm after thawing. In our study, it was observed that CLC did not alter the intracellular ROS level. However, it exhibited a positive impact on spermatological parameters, potentially attributed to its ability to enhance membrane stabilization by preserving sperm membrane integrity.

Several studies have reported synergistic effects when CLC was combined with compounds possessing different cryoprotective properties, leading to improvements in spermatological parameters after thawing (Inanc et al. 2019; Benhenia et al. 2016; Elsheshtawy 2024; Baishya et al. 2018; Li et al. 2020). On the other hand, some perspectives suggest that CLC reduces sperm membrane fluidity and permeability, thereby preventing substances that affect the sperm membrane structure from penetrating, which ultimately diminishes their effectiveness (Fayyaz, Ahmad, and Ahmad 2017). In the present study, the positive effect of the RES + CLC combination on spermatological parameters compared to the control and RES groups, along with its lack of superiority over the CLC group, indicates that RES and CLC do not exhibit a synergistic effect in ram sperm cryopreservation. The improved performance of the RES + CLC group compared to the control is likely attributed to the presence of CLC.

In conclusion, CLC had an adverse effect on the progressive motility of ram sperm during the pre‐freezing period, with this negative impact observed within 15 min of adding CLC to the sperm. However, it was determined that CLC provided protection against damage during the freezing and thawing of ram sperm. On the other hand, 20 µg/mL RES did not demonstrate any beneficial effects on spermatological parameters. Although the RES + CLC combination was found to be more successful than the control group, no synergistic effect was observed. Future studies on the cryopreservation of ram sperm could explore different doses and application methods of RES as well as investigate the timing of CLC treatment on sperm before the freezing process. These investigations could provide further insights into optimizing sperm preservation techniques.

## Author Contributions

**Eser Ahmet, Arıcı Ramazan, Yağcıoğlu Selin, Sandal Asiye İzem, Ersoy Nur, Demir Kamber, Ak Kemal**: conceptualization, methodology, data curation. **Eser Ahmet, Yağcıoğlu Selin, Evecen Mithat, Ak Kemal**: writing–review and editing. All authors have read and agreed to the published version of the manuscript.

## Ethics Statement

The authors confirm that the ethical policies of the journal, as noted on the journal's author guidelines page, have been adhered to and the appropriate ethical review committee approval has been received. The permission for the study was approved by the Istanbul University Animal Experiments Local Ethics Committee (04.01.2018‐5229).

## Conflicts of Interest

The authors declare no conflicts of interest.

### Peer Review

The peer review history for this article is available at https://publons.com/publon/10.1002/vms3.70172.

## Data Availability

All data generated or analysed during this study are included in this article.
